# Supplementary material for: What are the experiences and psychosocial needs of female survivors of domestic violence in Afghanistan? A qualitative interview study in three Afghan provinces
Source: BMJ Open. 2024 Jun 5;14(6):e079615. doi: 10.1136/bmjopen-2023-079615 (PMC11163623; doi:10.1136/bmjopen-2023-079615)
Supplement: Supplementary data [file bmjopen-2023-079615supp001.pdf]

UCL

Institute for Global Health

INTERVIEW QUESTIONNAIRE FOR SERVICE PROVIDERS

Title of Study: NIHR Global Health Research Group: A package of care for the mental health of survivors of violence in South Asia

[*This questionnaire is intended for REC purposes. The format of the interviews with service providers is qualitative and semi-structured, and as such may be adapted during the interview process to ensure a natural flow of conversation*.]

1. **Consent procedures and information about the research study**
- *Describe the project's purpose*
- *Describe how the research team will ensure the anonymity of the participants*
- *Read through each of the consent form questions with the participant to ensure understanding and have them sign the form/ give their explicit consent (recorded)*

2. **Violence and mental health**
- What kinds of violence do you see among women and children in your work?
- Do women and children who experience violence have more difficulties with mental health issues? In what ways?
- How are these issues currently addressed?
- What more could be done? How do you think these issues should be addressed?

3. **Conflict and mental health**
- Do you think the current conflict influences people's mental health in Afghanistan? In what ways?
- Have people had to leave their homes as a result of the conflict? Do you think this has impacted their mental health? In what ways?
- Who should be playing a role in addressing the mental health issues arising from the conflict, or people's experiences of violence during conflict?
- How are these issues currently addressed?
- What more could be done? How do you think these issues should be addressed?

4. **Mental health service provision**
- What kinds of mental health issues are you seeing among women or children who have experienced violence? What services do you offer these individuals?
- Are there considerations that need to be made for women or children that are different from men?
- Are there any gaps in the services that are available? What more could be done to address the mental health needs of women and children experiencing violence?

5. **Community responses to mental health**
- What happens at the community level when people experience mental health issues?

Institute for Global Health

- Do you think that communities should play a role in preventing or responding to violence/ mental health? If so, what kind of role do you see them playing?
- Who in the community do you think is best placed to respond to the violence/mental health needs of women and children?

6. **Closing**
- Is there anything that I haven't asked that you think I should know about this issue?
- Thank you for participating.
- *Discuss how the findings will be disseminated back to the participant.*
- *Ask if there's anyone else that the research team should speak to.*
