# Supplementary material for: What are the experiences and psychosocial needs of female survivors of domestic violence in Afghanistan? A qualitative interview study in three Afghan provinces
Source: BMJ Open. 2024 Jun 5;14(6):e079615. doi: 10.1136/bmjopen-2023-079615 (PMC11163623; doi:10.1136/bmjopen-2023-079615)
Supplement: Supplementary data [file bmjopen-2023-079615supp002.pdf]

UCL
Institute for Global Health

INTERVIEW QUESTIONNAIRE FOR ADULTS AND ADOLESCENTS

**Title of Study:** NIHR Global Health Research Group: A package of care for the mental health of survivors of violence in South Asia

[*This questionnaire is intended for IRB purposes. The format of the interviews with survivors of violence and their families is qualitative and semi-structured, and as such may be adapted during the interview process to ensure a natural flow of conversation*.]

1. **Consent procedures and information about the research study**
- *Describe the project's purpose*
- *Describe how the research team will ensure the anonymity of the participants*
- *Read through each of the consent form questions with the participant to ensure understanding and have them sign the form/ give their explicit consent (recorded)*

2. **Conflict and mental health**
- How does the current conflict impact on your daily life?
- Has the conflict had an impact on you in the past, and if so, how?
- Have you ever needed to leave your home because of the conflict? When, why and what impact did that have on you and your family?
- Have you ever experienced stress or anxiety as a result of the conflict? If so, in what ways?
- Did you seek help from someone for any stress or anxiety you may have experienced?
- Who did you speak to? How did they react?
- If you were to experience stress or anxiety in the future, who would you speak to about it?
- Is there anyone in your community you could speak to about such things?

3. **Personal experiences of violence and mental health**
- Have you ever witnessed violence either at home or outside of the home?
- If so, what happened? What caused the violence? How did you react?
- Did you go to anyone for help?
- How did you feel after witnessing the violence? Did you talk to anyone about how you were feeling?
- Has anyone close to you ever died because of an act of violence? When, why, how did this happen?
- Have you ever been slapped, hit, punched, or kicked by someone else? If so, what happened? Why do you think this happened? Did you tell anyone about it? How did they react?

4. **Mental health service provision**
- What services are you aware of that help people with stress, anxiety or depression?
- Would you use any of these services for yourself or a family member? Would you recommend them to others? Why or why not?

Institute for Global Health

5. **Community-based mental health responses**
- What do you think ‘good’ mental health means? What do you think it means to others in your family/ community?
- How do members of your community perceive mental health?
- If you or a family member were experiencing stress, anxiety or depression would you talk to someone about it? If so, who would you talk to? Would you go to a member of your community for advice?
- Have you known someone in your family or community experiencing mental health issues? If so, what did they do? Did they talk to someone about it? What happened?

6. **Closing**
- Is there anything that I haven’t asked that you think I should know about this issue?
- Thank you for participating.
- *Discuss how the findings will be disseminated back to the participant.*
- *Ask if there’s anyone else that the research team should speak to.*
